# Evaluation of a low-dose protocol for cone beam computed tomography of the temporomandibular joint

**DOI:** 10.1259/dmfr.20190495

**Published:** 2020-04-09

**Authors:** Durer Iskanderani, Mats Nilsson, Per Alstergren, Xie-Qi Shi, Kristina Hellen-halme

**Affiliations:** 1 Department of Oral and Maxillofacial Radiology, Faculty of Odontology, Malmö University, Malmö, Sweden; 2 Department of Oral and Maxillofacial Radiology, Faculty of Dentistry, King Abdulaziz University, Jeddah, Saudi Arabia; 3 Department of Medical Radiation Physics, Skåne University Hospital, Malmö, Sweden; 4 Department of Orofacial Pain and Jaw Function, Faculty of Odontology, Malmö University, Malmö, Sweden; 5 Division of Oral Maxillofacial Radiology, Department of Clinical Dentistry, Faculty of Medicine, University of Bergen, Bergen, Norway; 6 Section of Oral Diagnostics and Surgery, Department of Dental Medicine, Karolinska Institutet, Huddinge, Sweden

**Keywords:** Cone-beam CT, Radiation dosage, TMJ

## Abstract

**Objective:**

Evaluation of cone beam CT (CBCT) examination with a low-dose scanning protocol for assessment of the temporomandibular joint (TMJ).

**Methods:**

34 adult patients referred for CBCT imaging of the TMJ underwent two examinations with two scanning protocols, a manufacturer-recommended protocol (default) and a low-dose protocol where the tube current was reduced to 20% of the default protocol. Three image stacks were reconstructed: default protocol, low-dose protocol, and processed (using a noise reduction algorithm) low-dose protocol. Four radiologists evaluated the images. The Sign test was used to evaluate visibility of TMJ anatomic structures and image quality. Receiver operating characteristic analyzes were performed to assess the diagnostic accuracy. κ values were used to evaluate intraobserver agreement.

**Results:**

With the low-dose and processed protocols, visibility of the TMJ anatomical structures and overall image quality were comparable to the default protocol. No significant differences in radiographic findings were found for the two low-dose protocols compared to the default protocol. The area under the curves (A_z_) averaged for the low-dose and processed protocols, according to all observers, were 0.931 and 0.941, respectively. Intraobserver agreement was good to very good.

**Conclusion:**

For the CBCT unit used in this study, the low-dose CBCT protocol for TMJ examination was diagnostically comparable to the manufacturer-recommended protocol, but delivered a five times lower radiation dose. There is an urgent need to evaluate protocols for CBCT examinations of TMJ in order to optimize them for a radiation dose as low as diagnostically acceptable (the as low as diagnostically acceptable principle recommended by NCRP).

## Introduction

In recent decades, cone beam computed tomography (CBCT) has become an essential examination tool in oral and maxillofacial radiology. CBCT produces multiplanar images of high spatial resolution, which for many diagnostic tasks give information that is unattainable with two-dimensional projection radiography. In the diagnosis and treatment planning of patients with temporomandibular disorders (TMD), CBCT plays an effective role in assisting the clinicians when performing bony changes assessments with high diagnostic accuracy.^1^ In comparison to medical CT, no significant difference in diagnostic accuracy was found between the two techniques; moreover, it is a cost- and dose-effective alternative.^2,3^ Hence, it is considered as an appropriate imaging technique for evaluating bony changes of the temporomandibular joint (TMJ).^4^ However, the growing use of CBCT, partly because of its availability and easiness of use, demands a critical evaluation of the relatively high patient radiation dose. Many different scanning protocols are available, and sometimes the urge from clinicians to have more or less noise free images can increase the radiation dose to patients without adding more information to the specific aim of the investigation. Thus, with the many CBCT scanners on the market today, clinicians together with medical physicists should ensure that the scanner they are using is being set for the lowest possible radiation dose, yet capable of providing acceptable image quality for the particular diagnostic task.^5^

In current practice, most clinicians use the manufacturer-recommended exposure settings for the scanner in all clinical applications. This practice usually produces acceptable image quality, but might raise questions concerning the associated radiation dose. Attempts to optimize the patient radiation dose without sacrificing diagnostic outcome include approaches, such as optimizing tube current (mA), tube voltage (kV), and voxel size, selecting field of view (FOV) suitable to the diagnostic task, and using 180° or 360° of rotation.^6–11^

One practical approach of dose optimization is to adjust the scanner’s tube current to a level that the dentist may obtain enough diagnostic information of a given task at the expense of acceptable loss of image quality.^11,12^ The structures in the TMJs have the advantage of appearing in relatively high contrast on CBCT scans, and are thus less susceptible to a reduction in exposure than for low-contrast structures. The possibility to establish if a degenerative disease is present or not may be easier to establish, in comparison to a thin periodontal ligament for endodontic questions. Radiographic images of the TMJ do not have to be very sharp with a high signal to noise ratio. Moreover, advanced denoising algorithms may further improve the image quality of scans made using a low-dose protocol.

There is clearly a need for a broader understanding of reduced-dose protocols related to subjective image quality in specific dental CBCT applications. Such protocols have already been assessed for some dental situations but not in the context of TMJ imaging *in vivo*. The aim of this study was thus to evaluate a low-dose protocol with and without image processing in CBCT examination of the TMJ.

Our hypothesis was that the low-dose protocol differed significantly compared to the default, manufacturer-recommended protocol in regard to the radiographic diagnostics on TMJ.

## Methods and materials

The regional ethics review board in Lund, Sweden, approved this study (Dnr 2017/434). The study enrolled 34 adult patients (5 males and 29 females with a mean age of 57 years, ranged from 20 to 74 years) who had been referred for CBCT examination of the TMJ from the Department of Orofacial Pain and Jaw Function at Malmö University, Malmö, Sweden. For a power of 0.8 with a significant level of 0.05, around 60 TMJs were required. The indication to perform a CBCT investigation of TMJ was related to the clinicians’ questions after thorough history and clinical investigation. The main reason for the CBCT investigation was when the performed treatment failed. All participants underwent two consecutive CBCT examinations with different exposure settings. All study participants signed informed-consent forms. This form included information about the additional radiation dose, 20% extra, which is equivalent to less than one week of background exposure in Sweden.

### Cone beam CT examination

Two CBCT examinations (Veraviewepocs 3D F40, J. Morita Corp., Kyoto, Japan) were performed both TMJs of each patient which resulted in a total number of 68 CBCT volumes. The applied exposure protocols were: the default, manufacturer-recommended protocol (exposure settings: 90 kV, 5 mA, and 9.4 s) and a low-dose protocol (90 kV, 1 mA and 9.4 s). All other parameters were kept constant throughout the examinations, *i.e*. FOV of 40 × 40 mm, 180° rotation, and head position with the Frankfurt plane paralleled and the mid-sagittal plane perpendicular in relation to the floor of the mouth. Patients were informed to sit still during exposure. Multiplanar data were reconstructed with a pixel size of 0.125 mm, 1 mm slice thickness, and 1 mm slice interval.

### Image evaluation

**Figure 1.**
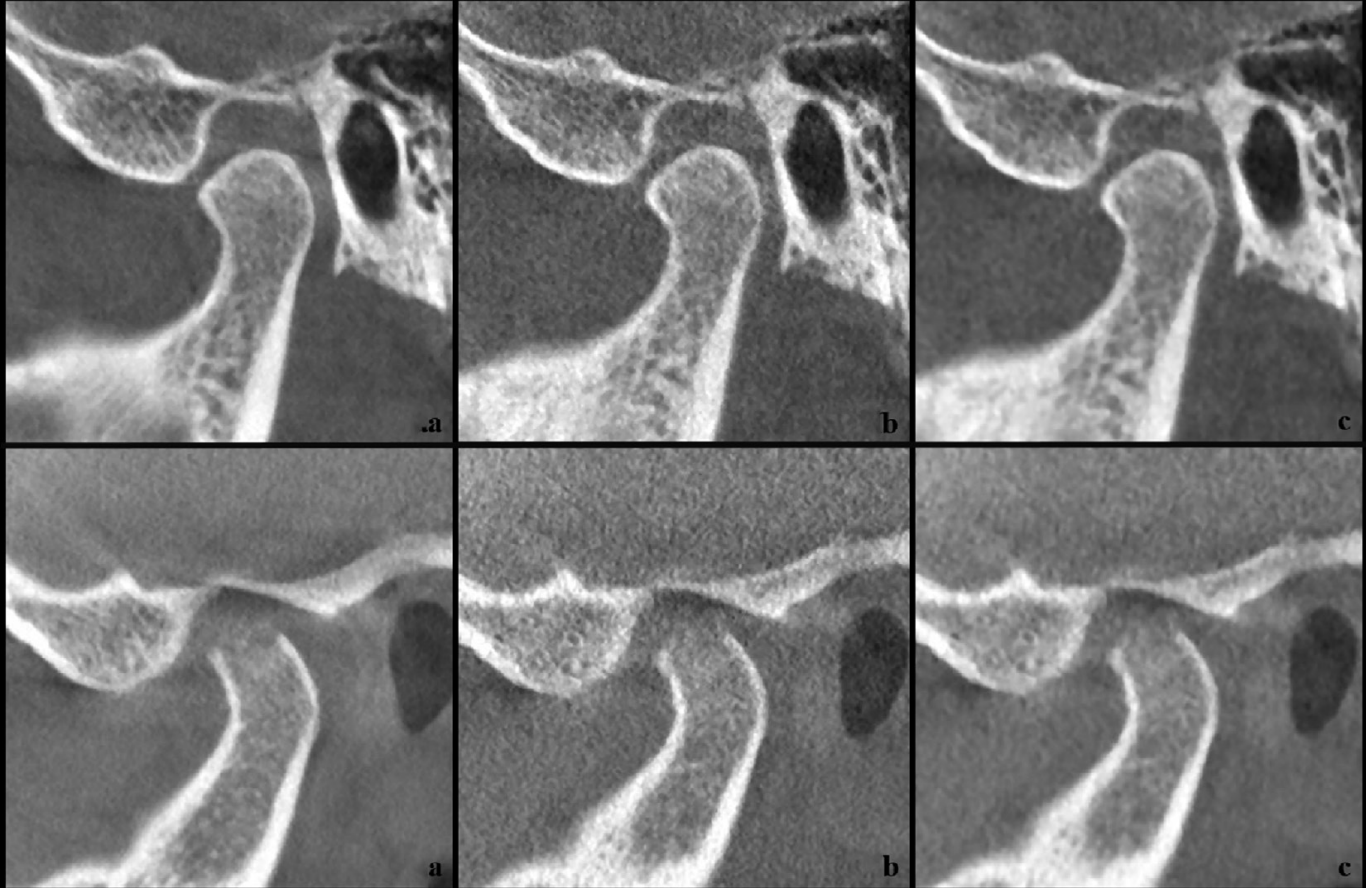
Examples of cone beam CT images for two temporomandibular joints using the three protocols (a) default, manufacturer recommended, (b) low-dose and (c) processed low-dose. 1 (a, b, c): case with no signs of degenerative joint disease; 2 (a, b, c) case with signs of degenerative joint disease.

68 reconstructed sagittal volumes were saved in DICOM format. These volumes were grouped into three stacks for display and processing using Image J (National Institutes of Health, Bethesda, MD): a default protocol stack, a low-dose protocol stack, and a processed low-dose stack with noise reduction (Figure 1). The processed stacks were created using an advanced noise reduction algorithm.^13^

We anonymized, randomly coded, and stored 204 data sets using Microsoft Excel Worksheet (Office Professional Plus 2016, Microsoft Corporation, Redmond, WA). Four (three senior and one junior) oral and maxillofacial radiologists evaluated all images. Before the study began, observer calibration was done on CBCT sagittal volumes of the first 25 stacks with a consensus on the instructions for assessing the images. The observers were blinded to any clinical or radiographic information and independently assessed the visibility of five anatomical structures: the outlines of the condyle, the articular eminence, and the articular fossa and the trabecular patterns of the condyle and the temporal bone. All observers used the same type of viewing monitor, (Barco MDCC-6430, 8500 Kortrijk, Belgium) under the same viewing conditions with no time limitations. The observers were allowed to adjust brightness and contrast when necessary. A 3-point scale was applied to assess the visibility of each anatomical structure: 1 = definitely visible, 2 = questionably visible, and 3 = not visible. Furthermore, the observers were asked to an overall impression on subjective image quality ranked as: 1 = diagnostically acceptable, 2 = diagnostically questionable, and 3 = not diagnostically acceptable. To assess the radiographic finding, the observers were asked to record their level of confidence about the presence of degenerative joint disease (DJD) and were stated as 1 = definitely not, 2 = probably not, 3 = questionable, 4 = probably and 5 = definitely according to Diagnostic Criteria of TMD (DC/TMD).^14^ Intraobserver agreement was determined by asking each observer to re-evaluate 40 TMJs at an interval of at least 14 days and under the same conditions.

### Statistical analysis

The five anatomical structures were pooled together in all settings for each observer and compared pairwise. For instance, the amount of “definitely visible” in the default protocol in relation to the number of “definitely visible” in the low-dose protocol. The overall image quality was evaluated in the same way. The results were analyzed using the Sign test^15^ with the level of significance at 0.05.

The default protocol of the CBCT unit was considered to be the reference standard to establish if DJD was present or not, by two observers (XS, KHH). A consensus was reached in cases of disagreement. Receiver operating characteristic (ROC) curves were used to analyze the radiographic findings concerning the presence of DJD for all the observers.^16^ The area under the curves (A_z_) were calculated. Intraobserver agreement was estimated using κ (κ) statistics.^17^ The values were interpreted according to the guidelines of Landis and Koch^18^ adapted by Altman.^15^

## Results

Visibility of the five anatomical structures and overall image quality, according to all observers, showed no difference (*p* = 0.00), for the two low-dose protocols compared to the default protocol. Only one observer found improvement in processing the images for both structures’ visibility and image quality between low-dose protocol and processed protocol, respectively (*p* = 0.79 (visibility), *p* = 1.00 (image quality)).

The evaluation of the default protocol for the 68 TMJ cases showed that half of them were sound while the rest had DJD with varying osseous changes. When establishing the reference standard, the two observers disagreed in one of the cases, which had minor erosions on the superior surface of the condyle.

**Figure 2.**
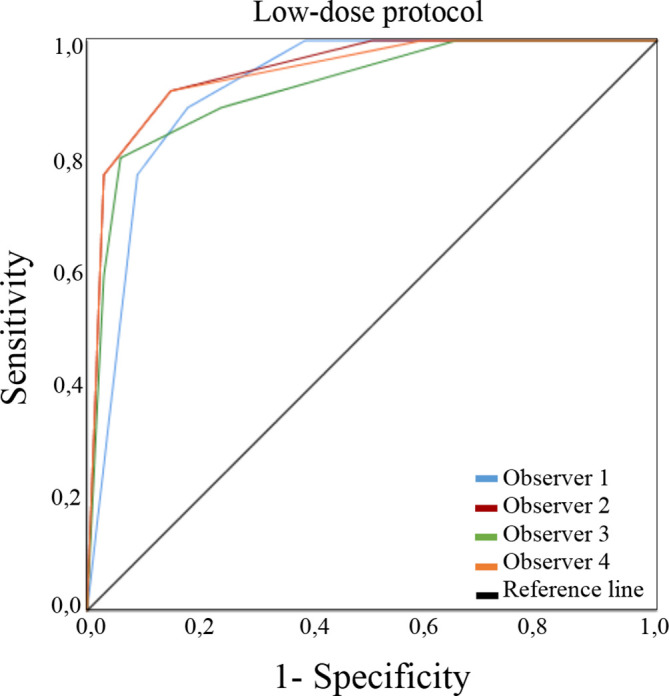
ROC curves for the low-dose protocol assessed by four observers according to the radiographic finding of degenerative joint disease. ROC, receiver operating characteristic.

**Figure 3.**
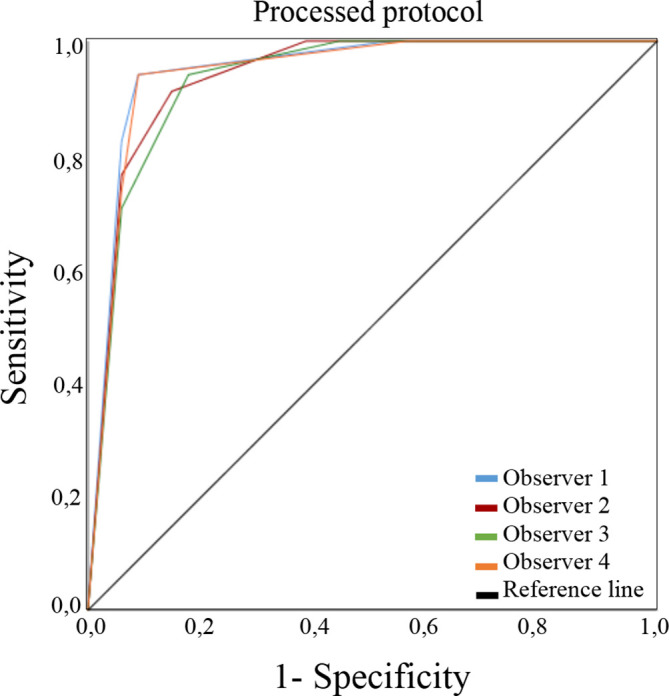
ROC curves for the processed protocol assessed by four observers according to the radiographic finding of degenerative joint disease. ROC, receiver operating characteristic

ROC curves for the low-dose and processed protocols assessed by all the observers according to DJD presence are presented in Figures 2 and 3, respectively. Table 1 presents the different A_z_ for each observer. The differences in the radiographic findings for the two low-dose protocols compared to the default one, as well as between them, were not significant. The average A_z_ for the observers were 0.931 for low-dose protocol and 0.941 for the processed protocol. Intraobserver agreement among the radiographic finding were good to very good for all observers (κ values = 0.75, 0.80, 0.80, 0.85).

**Table 1.** Area under the receiver operating characteristic curves (A_z_) for assessing the presence of the degenerative joint disease, according to four observers as evaluated on cone-beam computed tomography images produced by three protocols: the low-dose (1 mA tube current) protocol, the processed (low-dose with noise reduction) protocol, and the reference standard (“default” manufacturer-recommended, 5 mA tube current) protocol

	Low-dose protocol			Processed protocol		
	A_z_	Standard error	*p*-value	A_z_	Standard error	*p*-value
**Observer 1**	0.918	0.036	0.000	0.949	0.029	0.000
**Observer 2**	0.947	0.026	0.000	0.939	0.030	0.000
**Observer 3**	0.917	0.034	0.000	0.933	0.032	0.000
**Observer 4**	0.943	0.028	0.000	0.944	0.030	0.000

## Discussion

Several studies in the literature have demonstrated that CBCT imaging exposed with low-dose protocol may provide diagnostically acceptable image quality for various dental indications.^6–11^ The present study assessed a low-dose CBCT protocol for TMJ imaging, where tube current was lowered to just 20% of the manufacturer-recommended level in a clinical setting. In this study, the ethics review board approved that the potential benefit to future clinical practice for CBCT examination of the TMJ overcomes the increased radiation burden (equivalent to less than 1 week of background exposure in Sweden) for the patients included in the study. Therefore, we used an ethically approved study design that has been tested with different radiographic examinations.^19–22^

It is well known that manufacturer-recommended exposure settings for the CBCT scanners designed for dentistry vary widely, which results in varying amounts of radiation doses. Tube current (mA) and exposure time (s) control the number of X-ray photons emitted; thus, higher mAs increases the measured signal from the sensor and decreases image noise. The mA setting is linearly proportional to radiation dose.^23^ Hence, when tube voltage and exposure time are constant, lowering the mA setting by 50% reduces the delivered dose by half and consequently the quantum noise in the resultant image will be higher. Pauwels et al^12^ reported minimal loss of image quality when reducing tube current compared to when reducing tube voltage. Another study by Sur J et al,^11^ confirmed the potential to reduce dose through a reduction in tube current.

In line with the recommendations^24,25^ for the use of any radiographic investigation, optimization should be done whenever possible in order to expose the patient to the lowest radiation dose according to the As Low As Diagnostically Acceptable (ALADA) principle, which is proposed as a variation of the acronym ALARA (as low as reasonably achievable) to emphasize the importance of optimization according to a given diagnostic task in medical imaging.^25^ High image quality in the sense of being noise free and having high resolution is not necessary in all clinical situations. Structural changes in the few anatomical structures that comprise the region of the TMJ are quite visible at exposure levels well below those giving the highest image quality. Usually, we try to find osseous changes when any form of DJD is suspected; thus, lower radiation dose protocols are possible. A high resolution might be needed for assessing fine pathological changes, however. Thus, the diagnostic task determines the level of image quality needed and, in turn, the degree of exposure parameter optimization.^5^ In our faculty, the demand for CBCT investigation of TMJ has decreased over time. Only patients where no explanation of their pain and no effect of treatment for TMD were found, and for those where other questions that possibly could be answered would be subject to a CBCT investigation. Thus, the CBCT examination of the TMJ were considered justified.

Our results demonstrated the ability of a low-dose protocol to image the TMJ sufficiently well without significant difference in diagnostic accuracy. Thus, this protocol is preferable, since the radiation dose was 20% of the dose delivered using the default protocol. Kadesjö et al^26^ reported a 50% potential for dose reduction compared with the manufacturer’s recommendation for the CBCT examination of TMJ. Comparable results were found for reduced dose protocols with other diagnostic tasks.^6,10,11^

Reduction in dose is associated with degradation of image quality, principally due to the increased noise level. Noise influences both contrast resolution and spatial resolution and consequently, a representation of an object. However, in a sense of visual perception, the radiologist may still be able to see the object details and maintain diagnostic performance. When the diagnostic performance is influenced by a high level of noise, a noise reduction algorithm may be used to improve the image quality. Such programs filter out noise to varying degrees while preserving texture, contour, and fine details.^13^ The denoising method that we used was reported to be the technique of choice when very fine details are not required.^27^ We found that processing the images did not significantly improve the diagnostic accuracy (Table 1).

We used the default settings of the CBCT as a reference standard, although no “truth” can be obtained in this kind of clinical studies. We calculated the A_z_ in order to determine the validity of the two reduced-dose protocols for detecting the presence of DJD. Our findings revealed that both low-dose and processed protocols showed comparable diagnostic accuracy in relation to the reference standard (average A_z_ = 0.931 and 0.941, respectively), implying that the observers performance on detection of possible degenerative changes were comparable. A retrospective practice-based study found a similar result for pre-surgical implant assessment.^9^

It should be noted that these results were obtained in a real clinical setting, including factors such as motion artifacts that affect image quality. However, the limitations of this study could be that the results cannot be generalized to all CBCT machines. The number of observers chosen could be another limitation. However, the interobserver agreement was good, and therefore, further observers would, in our opinion, probably not contribute to any significant difference. A previous study by Hintze et al^28^ reported that the statistical power is dependent on the total number of evaluations, including the number of observers and surfaces evaluated for carious lesions, not on each separately. However, this kind of evaluation is known to show lower interobserver agreement due to the fact that these lesions are low contrast lesions. In comparison, TMJ structures appear with relatively high contrast.

As previous studies on low-dose protocols have also found adequate diagnostic image quality, our results could be considered together with these; it should be concluded that dose reduction achieved using a tube current below the recommendations of the manufacturer should always be investigated, in order to improve compliance with the ALADA principle.^25^ More important, a low-dose protocol should be the self-evident choice for younger patients, as the developing tissues of young patients are more radiosensitive and thus at a higher risk from X-ray exposure than adult tissues.^29^

## Conclusion

The hypothesis that low-dose protocol would affect the radiographic diagnostics on TMJ could be rejected, which means that for the CBCT unit used in this study, the low-dose CBCT protocol for TMJ examination was diagnostically comparable to the manufacturer-recommended protocol but delivered a five times lower radiation dose. There is an urgent need to evaluate protocols for CBCT examinations of TMJ in order to optimize them for a radiation dose as low as diagnostically acceptable (the ALADA principle recommended by NCRP).
